# A bifactor model supports unidimensionality of the International Knee Documentation Committee Subjective Knee Form in young active patients with anterior cruciate ligament tears: a retrospective analysis of a randomized controlled trial

**DOI:** 10.1186/s12955-023-02186-y

**Published:** 2023-09-12

**Authors:** Hana Marmura, Paul F. Tremblay, Alan M. J. Getgood, Dianne M. Bryant

**Affiliations:** 1 School of Physical Therapy, Faculty of Health Sciences, Western University, London, Canada; 2 Fowler Kennedy Sport Medicine Clinic, London, Canada; 3 Bone and Joint Institute, Western University, London, Canada; 4 Lawson Research, London Health Sciences Centre, London, Canada; 5 Department of Psychology, Faculty of Social Science, Western University, London, Canada; 6 Department of Surgery, Schulich School of Medicine and Dentistry, Western University, London, Canada; 7 Department of Health Research Methods, Evidence and Impact, McMaster University, Hamilton, Canada; 8 Faculty of Medicine, Michael G DeGroote School of Medicine, Hamilton, Canada

**Keywords:** Knee, Anterior cruciate ligament, Orthopaedic surgery, Youth, Patient reported outcome measures, Structural validity, Factor analysis, Symptoms, Function, Sport

## Abstract

**Background:**

The International Knee Documentation Committee Subjective Knee Form (IKDC) is the most highly recommended patient reported outcome measure for assessing patients with anterior cruciate ligament (ACL) injuries and those undergoing ACL reconstruction (ACLR) surgery. The IKDC was developed as a unidimensional instrument for a variety of knee conditions. Structural validity, which determines how an instrument is scored, has not been definitively confirmed for the IKDC in respondents with ACL injuries, and in fact an alternative two-factor/subscale structure has been proposed in this population. The purpose of this study was to determine the most appropriate structure and scoring system for the IKDC in young active patients following ACL injury.

**Methods:**

In total, 618 young patients deemed at high risk of graft rupture were randomized into the Stability 1 trial. Of the trial participants, 606 patients (98%) completed a baseline IKDC questionnaire used for this analysis. A cross sectional retrospective secondary data analysis of the Stability 1 baseline IKDC data was completed to assess the structural validity of the IKDC using exploratory and confirmatory factor analyses. Factor analyses were used to test model fit of the intended one-factor structure, a two-factor structure, and alternative four-factor and bifactor structures (i.e., a combination of a unidimensional factor with additional specific factors) of the IKDC, in a dataset of young active ACL patients.

**Results:**

The simple one-factor and two-factor structures of the IKDC displayed inadequate fit in our dataset of young ACL patients. A bifactor model provided the best fit. This model contains one general factor that is substantially associated with all items, plus four secondary, more specific content factors (symptoms, activity level, activities of daily living, and sport) with generally weaker associations to subsets of items. Although the single-factor model did not provide unambiguous support to unidimensionality of the IKDC based on fit indices, the bifactor model supports unidimensionality of the IKDC when covariance between items with similar linguistic structure, response options, or content are acknowledged.

**Conclusions:**

Overall, findings of a bifactor model with evidence of a reliable general factor well defined by all items lends support to continue interpreting and scoring this instrument as unidimensional. This should be confirmed in other samples. Clinically, based on these findings, the IKDC can be represented by a single score for young active patients with ACL tears. A more nuanced interpretation would also consider secondary factors such as sport and activity level.

**Trial registration:**

The Stability 1 trial for which these data were collected was registered on ClinicalTrial.gov (NCT02018354).

## Introduction

The International Knee Documentation Committee Subjective Knee Form (IKDC) is a region-specific 18-item questionnaire that collects information regarding symptoms, function, and sports activities, in patients with various knee conditions, reflected in one overall score [1]. Despite not being specific to anterior cruciate ligament (ACL) injuries, the IKDC has become the most highly recommended patient reported outcome measure (PROM) for assessing patients with ACL injuries and following ACL reconstruction (ACLR) surgery [2].

The IKDC results in little patient burden, with studies reporting adequate internal consistency reliability, test–retest reliability, construct validity, and responsiveness, with no floor or ceiling effects across patients with various knee conditions [2]. A main benefit of the IKDC is its purported unidimensionality and consequently the ability to extract one single score to compare across patients, time, and groups. This single score is intended to represent the underlying construct of “symptoms, function, and sports activity in subjects with impairment of the knee” [1]. Normative data and patient acceptable symptom states for the IKDC score have been reported to aid in outcome interpretation [2].

A systematic review of psychometric properties of PROMs used for ACL injuries indicated moderately positive evidence of test–retest reliability and hypothesis testing validity for the IKDC in this specific patient population [3]. However, there was no evidence to support the structural validity of the IKDC in an ACL patient population [3]. Structural validity is an important psychometric property of PROMs, often overlooked in clinical orthopaedic research. Structural validity refers to how well the score(s) of an outcome measure reflect the dimensionality of the construct(s) being measured [4]. This determines how an instrument should be scored. For example, poor structural validity might suggest that questionnaire subscales should be collapsed into larger general factors, or that distinct subscales should be separated out from a unidimensional score. Importantly, factor structure may differ across respondent populations and should therefore be confirmed within each population of interest prior to widespread use in that group.

Developers of the IKDC hypothesized that the underlying factor structure of the IKDC would be unidimensional, which was confirmed during its development through factor analysis with polychoric correlations, in a sample of patients with various knee conditions/injuries [1]. That analysis suggested one dominant factor (eigenvalue 9.03 for the first factor versus 1.76 and 1.19 for the next strongest factors) [1]. Consequently, the IKDC is reported as a single score for all respondents, despite a lack of studies confirming a unidimensional model.

Higgins and colleagues proposed an alternative shortened two-factor model of the IKDC, with 15 of the 18 original items, using over 1000 patients with various knee problems [5]. This structure was recently confirmed by a study of patients with ACL tears [6]. The two factors suggested in that model are 1) symptom and knee articulation and 2) activity levels [5, 6]. While these studies suggest two domains may be more interpretable and appropriate for scoring [5], the IKDC is still widely reported as a single score. Further, this proposed structure eliminates three questions in the IKDC concerning locking/catching of the knee, difficulty with running straight ahead, and difficulty with jumping and landing on the involved leg [5, 6]. We believe that these items, especially jumping and landing on the involved leg, are quite relevant to the young active ACL population, and therefore would like to confirm a valid structure of the IKDC that maintains these items for this group of respondents.

It is important to understand what scoring system (i.e., a single overall score, or two or more subscale scores), will be most meaningful and appropriate when interpreting IKDC outcomes in patients with ACL tears. These patients tend to be quite different than those with other knee conditions such as meniscal tears or osteoarthritis (often younger and more active in specific cutting/pivoting sports). Therefore it is possible that questionnaire structure might be different for these respondents, and that their outcome should be scored or interpreted differently. Based on the relative lack of evidence for structural validity of the IKDC for respondents with ACL injuries [2] and the possibility of an alternative structure [6], the purpose of this study was to determine whether the underlying factor structure of the IKDC is unidimensional or multidimensional in a sample of young active patients with ACL tears, and subsequently how the measure should be scored clinically for this population of interest.

We chose to focus on investigating structural validity at one time point, as the proposed structure should be confirmed in other datasets before tackling other psychometric properties reliant on the factor structure such as measurement invariance across time, internal consistency, or cross-cultural validity.

## Patients and methods

### Study design, setting and participants

This study was a retrospective secondary analysis of data obtained during the Stability 1 trial, a randomized clinical trial of young active patients undergoing ACL reconstruction [7].

Between January 2014 and March 2017, 1033 patients were screened for eligibility in the Stability 1 trial from nine centers (seven in Canada and two in Europe). Patients under 25 years old undergoing primary ACL reconstruction were included in the study if they were deemed at high risk of surgical failure and re-injury based on meeting at least two of the following criteria: a pivot shift grade 2 or higher, a desire to return to high-risk/pivoting sports, and/or generalized ligamentous laxity [7]. Based on this criteria, 367 patients were ineligible and 48 declined to participate. In total, 618 patients were randomized into the trial. Reconstruction procedures were performed using a hamstring tendon autograft, and patients were randomized to undergo reconstruction with or without lateral extra-articular tenodesis, a stabilizing procedure intended to reduce postoperative rotational knee laxity [7]. Patients were followed for two years postoperatively, with data collected for several patient-reported, functional, and clinical outcomes. A detailed study protocol and trial results are available elsewhere [7, 8].

Of the trial 618 randomized participants, 606 patients (98%) had complete baseline IKDC questionnaire data available for this analysis (2% missing data). Descriptive information on the entire Stability 1 sample is available in Table 1. Based on study inclusion criteria, the current dataset represents an appropriate sample to investigate the structural validity of the IKDC for our population of interest: young active ACL deficient patients. Baseline (post-injury, pre-surgery) data was used for this study and therefore the type of surgery performed was not relevant to these analyses. While no clear guidelines exist for determining sample size for factor analyses, samples of more than 500 participants are typically considered very good [9].

**Table 1:** Descriptive characteristics of patients included in the Stability 1 trial (*n* = 618)

Characteristic	Hamstring tendon autograft ACLR alone *N* = 312	Hamstring tendon autograft ACLR + lateral extra-articular tenodesis *N* = 306	*P* value
Sex, *n* males (%)	151 (48)	151 (49)	0.44
Age, years (mean ± SD)	18.8 ± 3.2	19.1 ± 3.3	0.33
BMI, kg/m^2^ (mean ± SD)	23.8 ± 3.7	24.0 ± 3.8	0.68
Time from injury to surgery, months (mean ± SD)	8.1 ± 18.9	9.3 ± 16.7	0.41
Operative limb, *n* dominant (%)	161 (52)	156 (52)	0.98
Mechanism of injury, *n* non-contact (%)	176 (74)	166 (72)	0.32
Sport played at time of injury, *n* (%)			
Soccer	100 (32)	122 (39)	0.06
Basketball	54 (18)	36 (12)	
Football or Rugby	54 (18)	56 (19)	
Downhill skiing	16 (5)	13 (4)	
Volleyball	19 (6)	12 (4)	
Other	66 (21)	66 (22)	
Graft source, *n* (%)			
Semitendinosus and gracilis tendons	301 (96)	297 (96)	0.57
Semitendinosus tendon	11 (4)	11 (4)	
Graft diameter, mm (median, min, max)	8 (6, 10)	8 (6, 10)	0.32
Chondral defect, ICRS grade > 3 any compartment, *n* (%)	15 (5)	14 (4)	0.52

*ACLR* Anterior cruciate ligament reconstruction, *BMI* Body mass index, *ICRS* International Cartilage Repair Society

### IKDC patient reported outcome measure

The IKDC is a knee-specific PROM consisting of 18 items with varying numbers of categorical responses (2, 5, and 11, Table 2). The items are summed and transformed to a single score from 0 to 100 (worst to best) representing the overall construct of “symptoms, function and sports activity” [1]. The varying question and response styles appear to form four distinct groups. Questions 2, 4 and 6 ask about patient’s knee pain, stiffness/swelling and locking/catching “during the past 4 weeks” with the addition of question 3 to capture the severity of pain. Questions 1, 5, 7, and 8 ask “what is the highest level of activity you can…” with the same five response options (ranging from unable to perform any activities to very strenuous activities). Question 10b similarly investigates activity level, asking about the participant’s current knee function on a 10-point scale ranging from “cannot perform daily activities” to “no limitation in daily activities”. Lastly, questions 9a to 9i ask “how does your knee affect your ability to…” with items 9a-f asking about lower intensity activities of daily living and items 9 g-i asking about higher intensity sport specific activities.

**Table 2:** Description of items and number of item response options in the International Knee Documentation Committee (IKDC) Subjective Knee Form

Item Number	Description	Response Options (number)
1	Activity level without pain	5
2	Frequency of pain	11
3	Severity of pain	11
4	Stiffness/swelling	5
5	Activity level without swelling	5
6	Locking or catching	2
7	Activity level without giving way	5
8	Activity level on regular basis	5
9a	Ascending stairs	5
9b	Descending stairs	5
9c	Kneeling	5
9d	Squatting	5
9e	Sitting	5
9f	Rising from chair	5
9 g	Running straight ahead	5
9 h	Jumping/landing on involved leg	5
9i	Stopping and starting quickly	5
10a (not included in score)	Knee function prior to injury	11
10b	Current knee function	11

The IKDC was administered electronically using software from EmPower Health Research Inc., and participants completed the questionnaire on an electronic tablet during their regularly scheduled clinic visit or at home from an electronic device of their choice. All questionnaires were administered and completed in English.

### Data analysis

All exploratory and confirmatory factor analyses were conducted with Mplus software, version 8.4 [10], using maximum likelihood (ML) estimation which is the default estimator in Mplus when variables are specified as continuous. We opted for this approach, instead of designating the items as ordinal (and using a WLSMV estimator) based on some recent research suggesting that continuous approaches may be better [11]. Based on questionnaire development, previous literature, and theoretical considerations, we planned to use exploratory factor analysis to test a unidimensional model, followed by a multidimensional model, with the number of factors selected based on a parallel analysis (a recommended procedure in EFA to identify the number of factors that should be extracted). Our rationale for using factor analysis rather than a Rasch modeling approach (used to confirm structural validity during the IKDC questionnaires development) was to investigate the dimensionality of the instrument beyond a single factor model. Rasch analysis or related item response models assume unidimensionality of the measure being investigated and in the case of the IKDC, we hypothesized that a unidimensional structure may not necessarily provide the best model.

In addition, we planned to use CFA to evaluate the fit of the multidimensional model obtained in EFA, not as a confirmation procedure but to see if the more restrictive model in CFA without cross-loadings would still provide a good fit. Finally, although not planned, we using a CFA approach to explore a four-factor model based on item content and style, and a bifactor model, allowing us to investigate a model that includes both a general factor and specific factors.

Model fit was assessed using χ^2^, root mean square error of approximation (RMSEA) and associated 90% confidence intervals (CI), comparative fit index (CFI), Tucker-Lewis index (TLI) and standardized root mean square residual (SRMR). The robust estimates of all indices were reported. Adequate fit was defined as CFI and TLI > 0.9, RMSEA and SRMR < 0.08 [12], factor loadings > 0.3 (indicating adequately strong correlation between items and their defined factor construct) and factor covariance < 0.85 (indicating that separate factors/constructs are adequately unique and should not be collapsed into one). Chi-square and the associated *p*-value were not used to formally assess fit because χ^2^ is sensitive to sample size and likely to reject the null hypothesis of good fit in any sample over 200 participants.

## Results

The single factor EFA of the IKDC revealed adequate factor loadings (> 0.3) for all items. However, the fit indices were not acceptable (χ^2^ (135) = 1762.04, *p* < 0.000, CFI = 0.673, TLI = 0.629, and RMSEA = 0.141 (90% CI: 0.135 to 0.147)). Although the first three unrotated factors had eigenvalues greater than one (6.92, 2.09 and 1.09 respectively), the parallel analysis suggested retaining two factors. The two factors suggested by the EFA (“symptoms” defined by items 2, 4, 6, 9a-i and “activity” defined by items 1, 5, 7, 8, 10) mirrored the two-factor model proposed by Higgins and colleagues (“symptoms and knee articulation” defined by items 2, 4, 6, 9a-i, 10 and “activity level” defined by items 1, 5, 7, 8, with items 6, 9 g, and 9 h eventually removed) [5]. The two factor EFA resulted in improved, but inadequate fit (χ^2^ (118) = 774.57, *p* < 0.000, CFI = 0.868, TLI = 0.829, and RMSEA = 0.096 (90% CI: 0.089 to 0.102)).

In the CFA analyses, we note that a one-factor model has the exact same fit as a one-factor model in EFA when the same estimator (ML) is used. To obtain an acceptable fit with the unidimensional model, 19 correlated residuals were required. With the large number of required modifications, this model is considered overfitted and unviable.

The initial fit indices of the CFA version of the two-factor model were also unacceptable in the current dataset (model 2, Table 3). Adequate factor loadings and fit indices were achieved only by adding multiple cross-loadings and correlated residuals. Including these modifications complicates the model and questionnaire scoring, which would not be practical in a clinical or research setting.

**Table 3:** Model structures tested the International Knee Documentation Committee Subjective Knee Form (IKDC) in young patients with anterior cruciate ligament tears using confirmatory factor analysis

	**Model Structure Tested**	χ^**2**^** (df)**	**CFI**	**TLI**	**RMSEA (90% CI)**	**SRMR**	**Covariances < 0.85?**	**Loadings > 0.3?**
1	One-factor	1762.04 (135)	0.673	0.629	0.141 (0.135 to 0.147)	0.091	N/A	Yes
2	Two-factor	982.65 (134)	0.829	0.805	0.102 (0.096 to 0.108)	0.072	Yes	Yes
3	Four-factor	450.72 (129)	0.935	0.923	0.064 (0.058 to 0.071)	0.055	Yes	Yes
4	Bifactor	291.78 (117)	0.965	0.954	0.050 (0.043 to 0.057)	0.047	Yes	Yes^a^

Acceptable values: CFI and TLI > 0.9, RMSEA and SRMR < 0.08, all factor covariances < 0.85, and all factor loadings > 0.3

All analyses were run with Mplus software using a continuous approach and maximum likelihood estimation

^a^Includes only loadings onto the general factor

The four-factor model tested was made up of the following factors: “symptoms” (items 2, 3, 4, 6), “activity level” (items 1, 5, 7, 8, 10b), “ADLs” (items 9a-f), and “sport” (items 9 g-i). The fit was adequate without modifications and improved from the one-and two factor models (model 3, Table 3). Correlations between the four factors in this model ranged from 0.42 to 0.73 and individual item loadings ranged from 0.36 to 0.87.

**Fig. 1:**
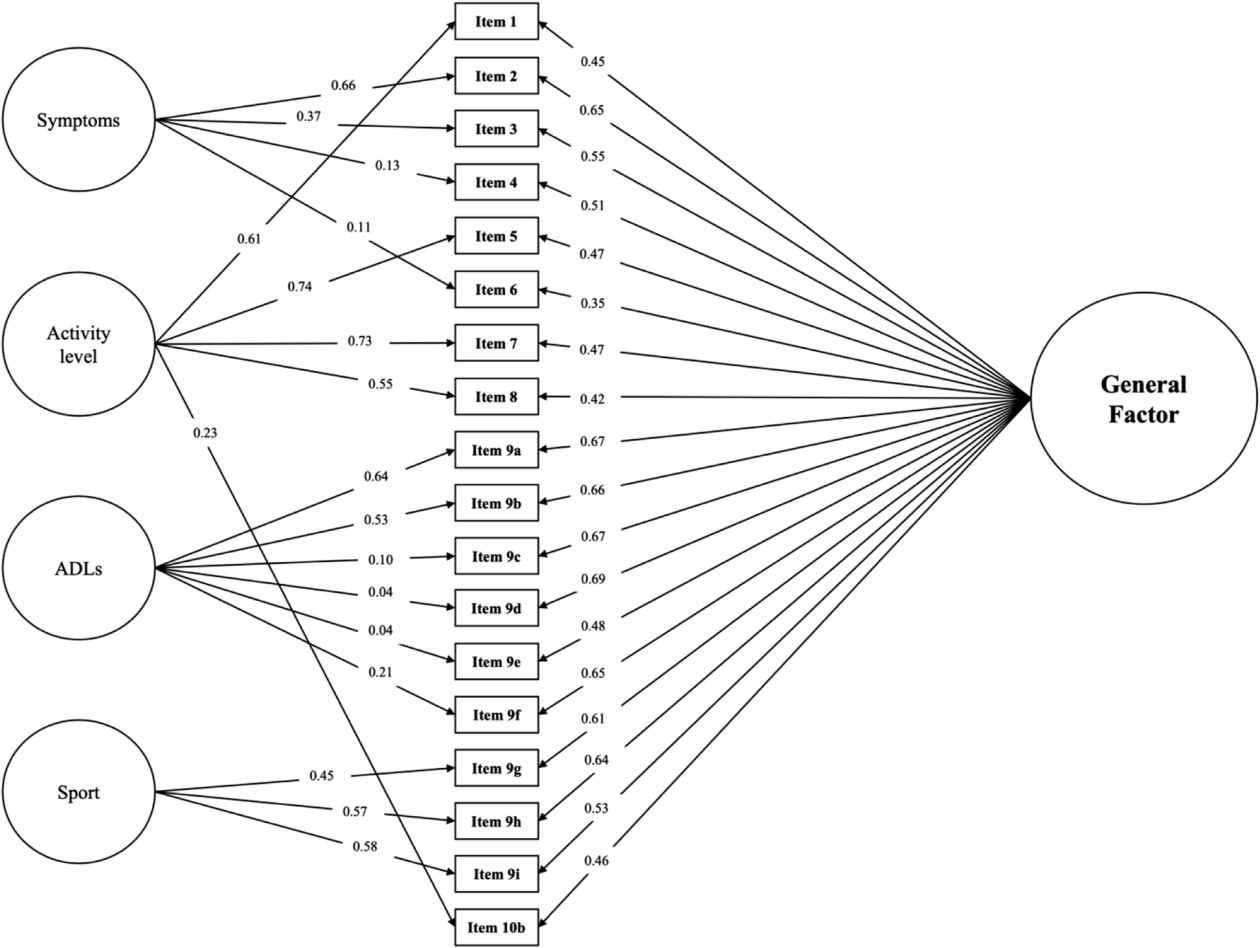
Path diagram with standardized factor loadings for the proposed bifactor structure of the International Knee Documentation Committee Subjective Knee Form (IKDC) in young patients with anterior cruciate ligament tears. The general factor would represent the construct of “symptoms, function and sports activities” as outlined during IKDC development. See Table 2 for descriptions of each questionnaire item. Standardized factor loadings displayed here are from the baseline patient dataset. ADLs; Activities of daily living

Model fit was further improved with a bifactor model of the IKDC including the four specific factors outlined above and a general factor (model 4, Table 3), with no modifications required. All items loaded adequately onto the general factor of the bifactor model (> 0.3). Of the specific factors, only sport had all items with loadings > 0.3, and activity level had only one item loading below this threshold (Fig. 1). The difference in χ^2^ values between the nested four-factor and bifactor models was 158.94, *p* < 0.001, indicating that the bifactor model has significantly better fit than the four-factor model. The optimal/final bifactor IKDC model achieved adequate fit and is visualized in Fig. 1.

Since the bifactor model was deemed most appropriate, bifactor model indices of reliability and unidimensionality were calculated and are outlined in Table 4. About 87% of the reliable variance in IKDC scores using the bifactor model can be attributed to the general factor (ω_R_) [13]. Approximately 12% of the reliable variance in scores would be attributed to the secondary factors (0.93–0.81; ω– ω_H_) and approximately 7% would be attributed to random error (1–0.93; 1-ω) [13]. The high ω_H_ and ω_R_ values for the general factor in this model (> 0.8), coupled with low ω_H_ and ω_R_ values for the secondary factors (0.14 to 0.61) suggests that the IKDC can be considered “essentially unidimensional” [13]. The factor determinacy score for the general factor was 0.922, while the scores for the secondary factors ranged from 0.814 (symptoms) to 0.898 (activity level). It is recommended that factor scores should only be utilized for individuals if the factor determinacy score is over 0.9 [14].

**Table 4:** Reliability and unidimensionality of the International Knee Documentation Committee Subjective Knee Form (IKDC) bifactor model in young active patients with anterior cruciate ligament tears

**Measure**	**Value**	**Description** [13]
**Omega (**ω**)**		
General	0.932	Estimate of internal reliability (analogous to Cronbach alpha for non-bifactor models)
Symptoms	0.716	
Activity	0.845	
ADLs	0.859	
Sport	0.860	
**Omega Hierarchical (**ω_**H**_**)**		
General	0.810	Proportion of systematic variance in total or subscale scores attributed to individual differences on the general factor or secondary factors, respectively
Symptoms	0.199	
Activity	0.377	
ADLs	0.124	
Sport	0.528	
**Relative Omega (**ω_**R**_**)**		
General	0.869	Proportion of reliable variance in the total scores due to the general factor, or reliable variance in subscale scores independent of the general factor
Symptoms	0.277	
Activity	0.447	
ADLs	0.144	
Sport	0.614	
**Factor Determinacy (FD)**		
General	0.922	Correlation between factor scores and the factors
Symptoms	0.814	
Activity	0.795	
ADLs	0.817	
Sport	0.898	
**Explained Common Variance (ECV)**		
General	0.584	Proportion of the common variance in total or secondary subscale scores explained by the general or secondary factors, respectively
Symptoms	0.062	
Activity	0.090	
ADLs	0.078	
Sport	0.187	
**Percent Uncontaminated Correlations (PUC)**	0.778	Proportion of covariance terms which only reflect variance from the general factor

*ADLs* activities of daily living

## Discussion

Overall, our analyses of several alternative models provide support for unidimensional scoring of the IKDC with some qualifications, in a population of young active patients with ACL tears.

Based on exploratory analyses of the IKDC, it appears as though two distinct factors may be present when used with ACL patients, in contrast to the intended unidimensional structure. However, eigenvalues from the exploratory analysis indicated similar strength of the first factor in this study and the initial IKDC development study [1] (6.92 and 9.03, respectively). Higgins and colleagues concluded that a two-factor model of the IKDC was most appropriate after using EFA, CFA, and item response theory to investigate data from 1517 patients with various knee problems [5]. The proposed two-factor structure included a symptom and knee articulation factor (items 2, 3, 4, 9a-df, 9i, 10b), and an activity level factor (items 1, 5, 7 and 8). This model was replicated in a recent study of 319 ACL patients using Bayesian structural equation modeling, which ultimately concluded poor structural validity of the IKDC in ACL patients [6]. While a very similar structure emerged in our own two-factor EFA of the IKDC, we felt that the grouping of items may be strongly related to similar linguistics and response options amongst questions, rather than distinct questionnaire constructs.

However, when confirmatory analyses were conducted, both the single and two-factor structure of the IKDC required too many modifications to obtain adequate fit for us to have confidence in the utility of these models for our patient population. Accepting the one- or two-factor structures of the IKDC with the required modifications would complicate scoring and convolute interpretability of IKDC in young active patients with ACL tears. The pattern of item correlations suggested by modification indices during the CFA process made it clear that items worded similarly or sharing the same number of response options tended to group together. This is intuitive and may indicate clustering of items based on linguistic factors rather than constructs of the questionnaire. This analysis outlines potential issues with multiple rating scales in the same unidimensional outcome as a consideration when developing future measures. When creating new orthopaedic PROMs, it may be helpful to standardize the response options and linguistic style of questions to avoid such item clusters.

There were four content factors created: symptoms (items 2, 3, 4, 6), activity level (items 1, 5, 7, 8, 10b), ADLs (items 9a-f), and sport (items 9 g-i), and a four factor model was tested based on these theoretical relationships between items. Finally, a bifactor model was run to test the hypothesis that there is indeed one overall general factor of “symptoms, function and sports activity” as intended, with residual associations between subsets of items similarities in with linguistics, response options, or content. The symptoms factor covers patients’ pain, stiffness, and locking/catching of the knee. The activity level factor consists of questions about the activity level a patient can participate in without various symptoms. The ADL and sport factors are distinct in the types of activities being inquired about. We believe that this distinction is important for our population of interest and a consideration that was missing from the previously proposed two-factor structure.

The four-factor and bifactor model showed acceptable fit indices without modifications, unlike the previously tested structures. The fact that some items’ loadings decreased when moving from the four-factor model without the general factor to the bifactor model suggests that these particular items overlap more strongly with the general factor. Additionally, the fit comparison of these nested models indicated improved fit with addition of the general factor (bifactor specification). Each IKDC item showed adequately strong (≥ 0.35) associations with the general factor, and generally weaker and inconsistent associations with the secondary, more specific content factors.

Importantly, measures of explained systematic, reliable, and common variance (ω_H_, ω_R_, and ECV, respectively) were adequately high for the general factor but were low for all four secondary factors. This suggests that while similarly worded items or those with similar content tended to group together, they did not necessarily represent distinct constructs that should form their own subscales. Additionally, only the general factor reached the threshold of factor determinacy (0.9) that would recommend its score be used. The score for the general factor is the total IKDC score currently in use. The secondary sport and activity level factors showed some evidence of reliability and adequacy as independent factors, however, the estimates are not substantial or consistent enough that we would advocate the use of these subscale scores. Taken together, the reliability indices suggest that the general factor stands largely on its own but that there may be value in looking at the sport and activity level factors, as some people may show differentiation between them (e.g., a high score on two and moderate score on the third). Overall, these analyses suggest that the IKDC can be deemed “essentially unidimensional”.

Therefore, in the context of young active ACL patients, we propose a bifactor model structure for the IKDC whereby one general factor best represents the items as intended, but item clusters caused by differences in content and item construction (wording and response options) are accounted for to obtain adequate fit. Clinically, this means that clinicians and researchers can continue to use the IKDC as it was designed, as a unidimensional instrument for young active patients with ACL tears. What the bifactor model adds is acknowledgement of item clusters depicting symptoms, activity level, ADLs, and sport. Clinicians and researchers can look at these clusters of items more closely to determine what secondary factor may be driving a high or low score, and patient progress or lack thereof. Importantly, these factors should not be treated as subscales, and scoring of the IKDC has been preliminarily validated in this population to remain as is. Forming subscales in addition to the total score creates a multi-factor correlated model, with resultant scores which would be highly correlated and redundant, because they would include a mix of both the general and secondary factors. The bifactor model therefore does not change use or scoring of the IKDC in young active patients with ACL tears but increases confidence in the utility of one total score and adds further nuance to the interpretability of the scale for these patients.

### Limitations

The factor structure identified in this study may be sensitive to the study sample. We used the entire sample for our analyses in an effort to maintain power and precision, however we cannot be confident in the robustness of the analyses, especially given the alternative structures shown in previous work with other datasets. Only baseline, and not post-operative data was used for this analysis. Assessing additional time points and the measurement invariance of the proposed structure across time is beyond the scope of the initial CFA of the IKDC covered in this paper. Therefore, we cannot be sure that the bifactor model and essential unidimensionality shown here would have adequate fit in other datasets at later timepoints following ACL injury. Analyses of measurement invariance in other datasets from patients following an ACL injury would be required to assess consistency in the outcome measure’s dimensionality for this specific population. Our analysis represents one proposed structure for the IKDC which should be confirmed in other datasets first before investigating other psychometric properties which rely on the assumption of structural validity such as internal consistency reliability and cross-cultural validity.

## Conclusion

Overall, the bifactor model lends support to continue reporting the IKDC as a unidimensional score for young active patients with ACL tears, recognizing the presence of specific secondary factors made up of items with similar formatting/content. Clinically, it appears that the IKDC can be administered and scored as the intended unidimensional questionnaire for young active patients with ACL tears, with secondary factors available for further interpretability of outcome scores.

## Data Availability

The datasets used and/or analysed during the current study are available from corresponding author on reasonable request.

## Abbreviations

IKDC: International Knee Documentation Committee Subjective Knee Form
ACL: Anterior cruciate ligament
ACLR: Anterior cruciate ligament reconstruction
PROM: Patient reported outcome measure
EFA: Exploratory factor analysis
ML: Maximum likelihood
CFA: Confirmatory factor analysis
RMSEA: Root mean error of approximation
CI: Confidence interval
CFI: Comparative fit index
TLI: Tucker-Lewis index
SRMR: Standardized root mean square residual
ADLs: Activities of daily living
